# Visit of the Shahzada to the St. Thomas's Hospital

**Published:** 1895-09-07

**Authors:** 


### VISIT OF THE SHAHZADA TO ST. THOMAS'S HOSPITAL.

We have all along felt that a visit to one of London's great hospitals should be included among the Shahzada's sightseeings, and no better choice could have been made than St. Thomas's Hospital, whose imposing exterior must have become well enough known to the Afghan visitors during their stay in London. Having seen so much of the warlike side of our national institutions, it was entirely as it should be that Nasrulla Khan should also carry away with him a more peaceful impression, in seeing the care bestowed upon the sick and suffering in our great city. We are glad, therefore, to be able to report the entire success of our efforts which induced the Shahzada on almost the last day of his stay in England to visit St. Thomas's Hospital. At half-past three on Saturday last he was received at the Westminster Bridge entrance by the Dean of the Medical School, Mr. G. H. Makins, the Steward, Mr. Walker, and Mr. E. M. Hardy. His Royal Highness was accompanied by Colonel Sir Adelbert Talbot, who acted as his interpreter, General Sir Michael Biddulph, Sir Acquin Martin, Surgeon-Major Leahy, Sirdar Mahomed Husan Khan, and Sirdar Mahomed Akran Khan.

The first visit was made to the Governor's Hall, from which so splendid a view of the river and the Houses of Parliament is to be had, and Nasrulla Khan was then conducted through St. Thomas's Home (where the system of patients' payments was explained to him), out on to the terrace, into the Nightingale Home, and through the Eye Ward, afterwards going into one of the operating theatres, where some of the principles of antiseptic surgery were explained to him, and where he examined the surgical instruments with interest. Then the Alexandra Ward (women's) was inspected, the Victoria (children's), and the Edward (male surgical). Very special interest was shown by the Shahzada in the dispensary, where he made quite a long stay, watching the making-up of various bottles of medicine. The hospital kitchen, too, was a source of keen interest, the gas being lighted and the working of the stoves explained. The registrar's room was next gone into, and the method of recording cases demonstrated. Nasrulla wished also to see the disinfecting apparatus, but as it had been recently in use this was not feasible. Then came the medical school, the club and museum, &c., the museum proving a particular attraction, the Shahzada asking many questions, the answers to some of which must have been more than a little difficult of interpretation. Shown the chemical department, Nasrulla inquired of what use that branch of study could be to medical students!

Taken altogether, his visit evidently pleased the Shahzada, and he made a little speech to that effect before finally taking his leave, also contributing £150 towards the Special Appeal Fund, and appearing much interested in the details of the foundation and endowment. Several of the Sisters were presented to him during his progress through the wards, where he showed a good deal of interest in the cases, and examined the plaster of Paris bandages, &c.

It would be interesting to know what sort of comparisons were drawn by Nasrulla and his Afghan companions, to whom he talked volubly throughout his visit, between this English hospital and that in Cabul, in which it will be remembered the Ameer takes much interest, partly no doubt through the influence of the English medical officers attached to his suite, Miss Hamilton and Dr. Leahy. Several visits have been paid by the latter, accompanied by an Afghan doctor, to St. Thomas's Hospital, and they were present on one occasion at an ovarian operation by Mr. Makins. Stimulated by his inspection of an English hospital, it may be that the Shahzada will follow his father's example and keenly interest himself on his return home in that in his native city of Cabul.

